# Ganglioside GM1 contributes to extracellular/intracellular regulation of insulin resistance, impairment of insulin signaling and down-stream eNOS activation, in human aortic endothelial cells after short- or long-term exposure to TNFα

**DOI:** 10.18632/oncotarget.23726

**Published:** 2017-12-15

**Authors:** Norihiko Sasaki, Yoko Itakura, Masashi Toyoda

**Affiliations:** ^1^ Research Team for Geriatric Medicine, Vascular Medicine, Tokyo Metropolitan Institute of Gerontology, Sakaecho 35-2, Itabashi-Ku, Tokyo 173-0015, Japan

**Keywords:** TNFα, vascular insulin resistance, GM1, aortic endothelial cell, aging

## Abstract

Vascular insulin resistance induced by inflammatory cytokines leads to the initiation and development of vascular diseases. In humans, circulating TNFα levels are increased during aging, suggesting a correlation between vascular insulin resistance and plasma TNFα levels. Currently, the precise molecular mechanisms of vascular insulin resistance mediated by TNFα are not well characterized. We aimed at clarifying whether glycosphingolipids contribute to vascular insulin resistance after inflammatory stimulation. In this study, we examined vascular insulin resistance using human aortic endothelial cells after treatment with different concentrations of TNFα for different time intervals for mimicking *in vivo* acute or chronic inflammatory situations. We show that ganglioside GM1 levels on cell membranes change depending on time of exposure to TNFα and its concentration and that the GM1 expression is associated with specific extracellular/intracellular regulation of the insulin signaling cascade. Furthermore, we provide evidence that factors such as aging and senescence affect the regulation of insulin resistance. Our data suggest that GM1 is a key player in the induction of vascular insulin resistance after short- or long-term exposure to TNFα and is a good extracellular target for prevention and cure of vascular diseases.

## INTRODUCTION

Vascular endothelial cells (ECs) constitute the endothelium of blood vessels, which forms an interface between blood and vessel walls and plays important roles in vascular homeostasis. Excessive activation or dysfunction of ECs is considered to lead to the development of vascular-related diseases, including restenosis, arteriosclerosis, and cancer [1]. Insulin signaling regulates important functions in ECs and contributes to the maintenance of vascular integrity. For example, nitric oxide (NO) production upon endothelial NO synthase (eNOS) activation (mediated by insulin signaling) maintains endothelial barrier integrity, leading to inhibition of accumulation and retention of atherogenic apolipoprotein B-containing lipoproteins in the subendothelial space. NO production also suppresses the expression of adhesion molecules on ECs, resulting in inhibition of monocyte and monocyte-derived macrophage accumulation and thus attenuating the progression of atherosclerosis, which accompanies EC injury caused from intracellular deposition of lipid droplets [2–8]. Vascular insulin resistance is therefore considered to play an important role in the pathogenesis of vascular and vascular-related diseases, including type 2 diabetes [8–10]. Inflammatory mediators potentially contribute to vascular insulin resistance [11]. It has been reported that circulating serum levels of the pro-inflammatory cytokine tumor necrosis factor-alpha (TNFα) are increased in obese and elderly people and that high serum levels of TNFα are associated with a high prevalence of vascular diseases [12, 13]. In high-fat diet-fed mice, phosphatase and tensin homologue upregulated by TNFα induces vascular insulin resistance [14]. Furthermore, production of TNFα by hypertrophic and senescent adipocytes is known to induce insulin resistance in ECs [9, 15]. Therefore, TNFα is a potential contributor to vascular insulin resistance in elderly people and/or people suffering from obesity. The precise molecular mechanisms of vascular insulin resistance induction via TNFα still remain to be elucidated, particularly in humans and at the cell surface level.

Glycosphingolipids are composed of a glycan structure attached to a lipid tail containing the sphingolipid ceramide. Glycosphingolipids expressed on cell membranes have frequently been used as developmental marker molecules and have been suggested to have important biological functions [16–18]. GM1, one of the gangliosides (molecules composed of glycosphingolipids with one or more sialic acids linked), is a well-known key component of lipid rafts. Changes in GM1 levels affect cell surface expression of raft-associated proteins and contribute to reduce membrane fluidity, leading to several cellular dysfunctions, such as impaired signal transduction [19–21]. It has been demonstrated that gangliosides are fine regulators of insulin signaling and that altered compositions of cell surface gangliosides often result in cellular responses associated with physio-pathological conditions [22, 23]. In 3T3-L1 adipocytes, increased levels of monosialodihexosylganglioside (GM3) upon TNFα stimulation were found to contribute to insulin resistance in pathological conditions such as obesity [24]. Furthermore, it was shown that potent inhibitors of glycosphingolipid synthesis improve ganglioside-mediated insulin sensitivity in mice [25, 26]. We previously demonstrated that increased GM1 levels associated with senescence and aging contribute to insulin resistance in ECs [27]. Thus, gangliosides play important roles in the induction of insulin resistance. It is still unknown, however, whether (and what kinds of) gangliosides contribute to insulin resistance in ECs after TNFα stimulation.

We aimed at clarifying the molecular mechanisms of induction of vascular diseases via inflammation in humans, but due to the high complexity of these mechanisms it is currently difficult to recapitulate the *in vivo* situation. For this purpose, we designed and performed *in vitro* experiments representing *in vivo* acute or chronic vascular inflammatory situations. We examined vascular insulin resistance using non-aged, aged or senescent human ECs after treatment with different concentrations of TNFα for different time intervals. In particular, we focused on gangliosides of ECs and we hypothesized that changes in the levels of cell surface gangliosides upon TNFα exposure contribute to insulin resistance in ECs. In this study, we demonstrate that GM1 is a key player in the regulation of intensity and duration of vascular insulin resistance by using *in vitro* models that mimic inflammation in aging humans.

## RESULTS

### GM1 levels increase in TNFα-treated human aortic ECs (HAECs)

**Figure 1:**
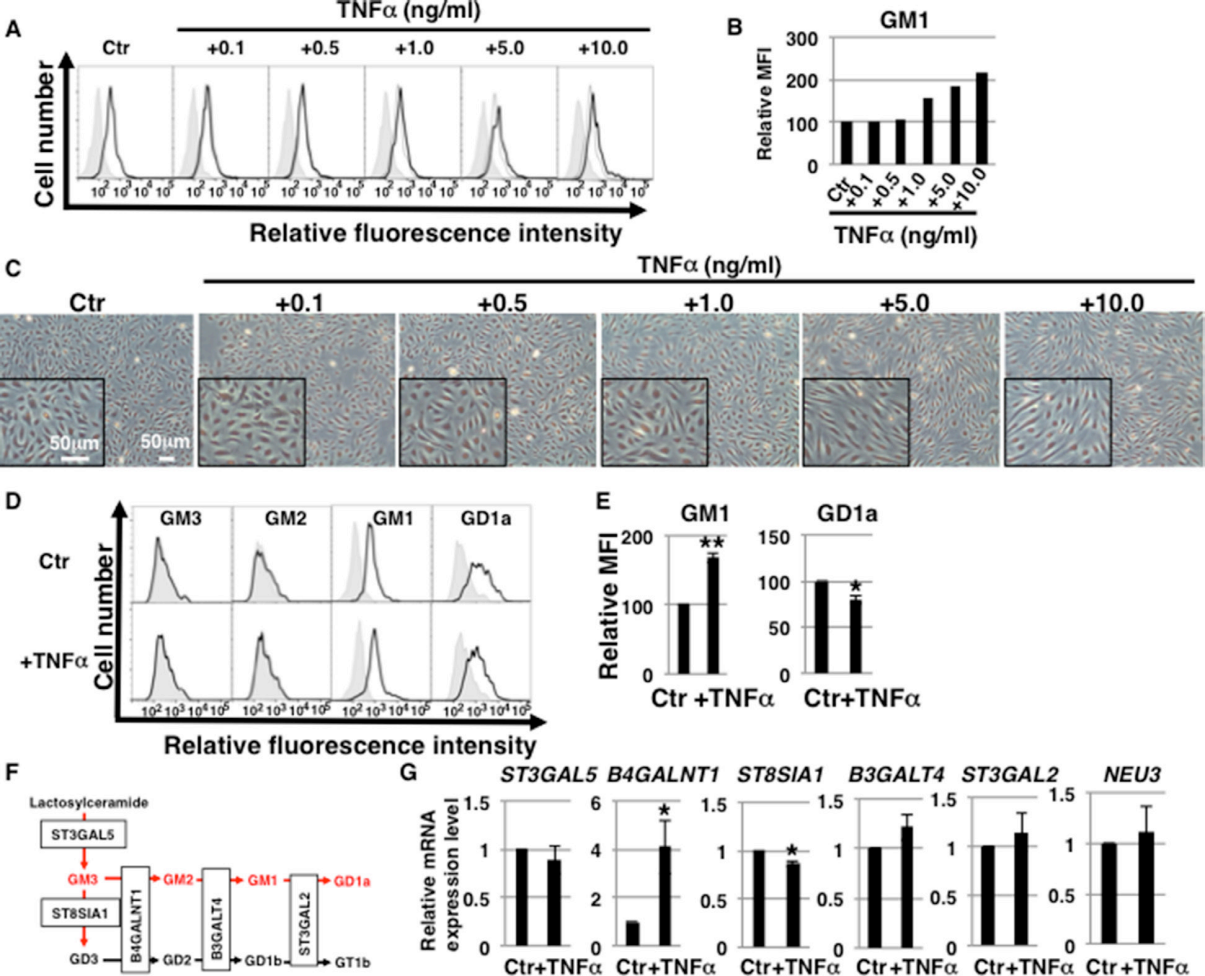
Ganglioside GM1 levels increase in TNFα-treated HAECs (**A**) Levels of GM1 in TNFα-treated HAECs analyzed by flow cytometry. Representative results are shown. GM1 expression in control cells and negative controls is depicted by *black dot lines* and *thin gray lines*, respectively. (**B**) Mean fluorescence intensities (MFIs) of GM1 expression relative to control HAECs. (**C**) Representative photos of HAECs 3 days after exposure to TNFα (0.1 ng/ml–10 ng/ml). Inset image is at high magnification. (**D**) Cell surface levels of gangliosides in short-term (3 days) 1 ng/ml TNFα-treated HAECs analyzed by flow cytometry using antibodies against each ganglioside. Representative results are shown. Negative controls are depicted by *thin gray lines*. (**E**) MFIs relative to control HAECs. Results are presented as means ± SD from three independent experiments. (**F**) Pathways of gangliosides and glycosyltransferases contributing to each synthetic pathway. Gangliosides highlighted in red fonts were examined in this study. (**G**) Real-time PCR analysis of the glycosyltransferases shown in (F) and *NEU3* using cDNA derived from control and short-term (3 days) 1 ng/ml TNFα-treated HAECs. The results are shown after normalization against values obtained for control HAECs (value = 1). Results are presented as means ± SD from three independent experiments. ^*^P < 0.05; ^**^P < 0.01. Control (Ctr): untreated cells.

The amount and composition of gangliosides in cell membranes can change depending on cellular conditions, as for example it was shown for senescent HAECs with increased levels of ganglioside GM1 [17, 18, 27]. It is unknown whether and what kinds of gangliosides are affected in ECs after TNFα stimulation. To study changes in cell surface GM1 levels in TNFα-stimulated ECs, we performed fluorescence-activated cell sorting (FACS) analysis of HAECs 3 days after incubation with several concentrations of TNFα (0.1 ng/ml–10 ng/ml). We found that expression of GM1 on cell surfaces increased in a concentration-dependent manner (above 1 ng/ml) (Figure 1A and 1B) and that the morphology of treated HAECs changed to spindle-shaped fibroblast-like in the presence of high concentrations of TNFα (above 5 ng/ml) (Figure 1C). Thus, 1 ng/ml TNFα induces changes in cell surface expression of GM1 without concomitant morphological changes. We next examined changes in the expression of the other three main gangliosides (GM3, GM2, GD1a) in 1 ng/ml TNFα-treated HAECs. FACS analysis revealed that GM3 and GM2 expression was undetectable; whereas GM1 and GD1a were mainly present on HAECs and that their levels changed in 1 ng/ml TNFα-treated HAECs (Figure 1D). As shown in Figure 1E, GM1 levels significantly increased in 1 ng/ml TNFα-treated HAECs compared with control cells, whereas GD1a levels decreased. Immunocytochemical staining confirmed these results (Supplementary Figure 1). To elucidate the mechanisms contributing to the observed increase in GM1 levels upon exposure to TNFα, we analyzed the expression levels of the glycosyltransferases involved in the ganglioside synthetic pathways (Figure 1F) and of sialidase (*NEU3*), which modulates ganglioside content by removing sialic acid [28]. Real-time PCR analysis showed that the expression of *B4GALNT1*, which catalyzes the synthesis of GM2 prior to GM1 synthesis, was significantly increased and that expression of *ST8SIA1*, which does not contribute to the GM1 synthetic pathway, was decreased in 1 ng/ml TNFα-treated HAECs compared with control cells (Figure 1G). These changes in glycosyltransferase expression are therefore likely to positively contribute to the GM2 and GM1 synthetic pathway, presumably resulting in the upregulation of GM1 in TNFα-treated HAECs.

### Increased GM1 levels contribute to insulin signaling reduction in 1 ng/ml TNFα-treated HAECs

**Figure 2:**
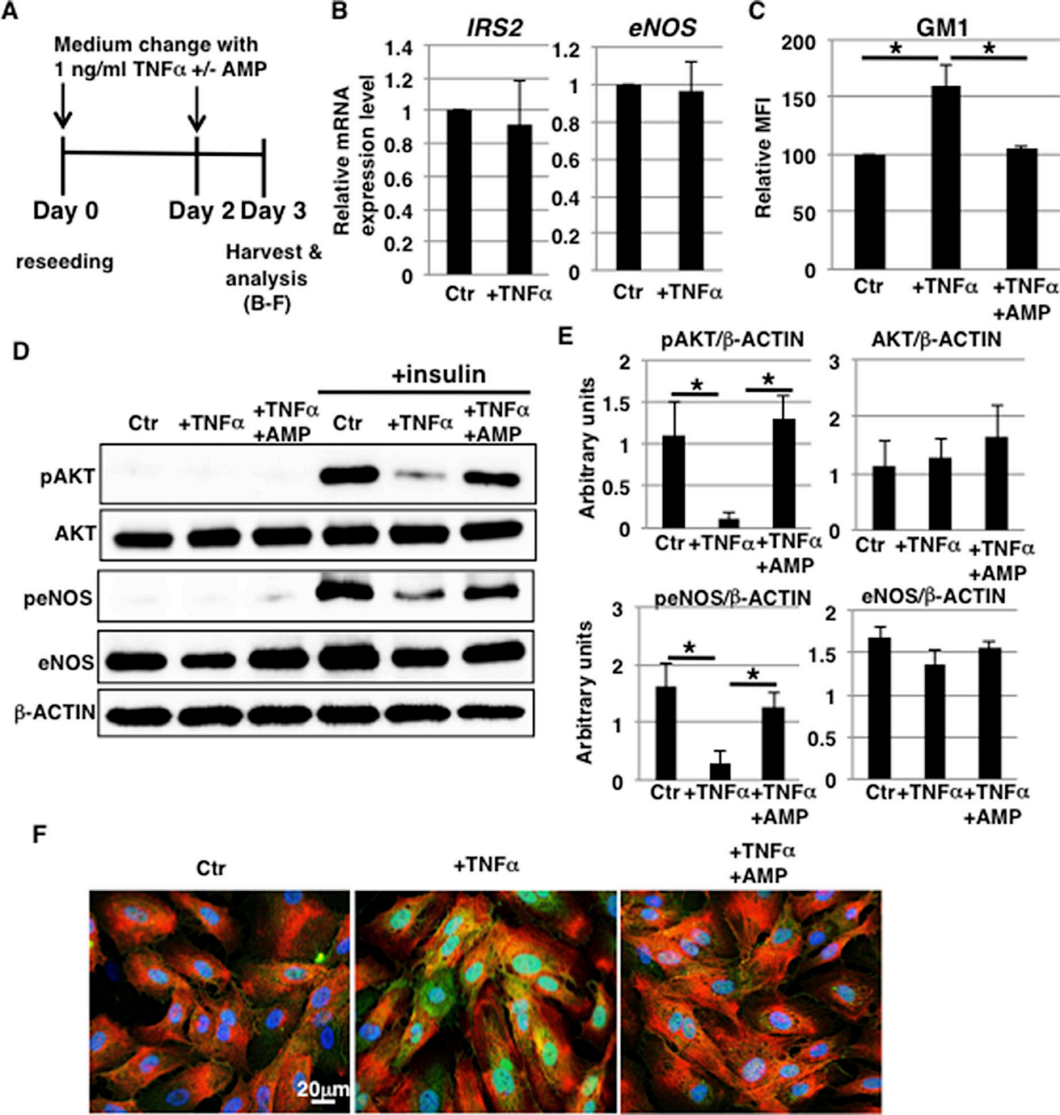
Increased GM1 levels in 1 ng/ml TNFα-treated HAECs reduce insulin signaling (**A**) At day 0, cells were reseeded with 1 ng/ml TNFα and with or without AMP-dNM in HAEC medium and incubated for 3 days. At day 3, cells were harvested and analyzed (**B**–**F**). Control cells were grown in HAEC medium. (B) Real-time PCR analysis of *IRS2* and *eNOS* performed using cDNA derived from control and TNFα-treated HAECs. Results shown were normalized against values obtained for control HAECs (value = 1). (C) Cell surface levels of GM1 in TNFα-treated HAECs with or without AMP-dNM treatment analyzed by flow cytometry. MFIs of three independent experiments relative to control HAECs are shown. (D) Western blot analysis of insulin signaling performed in TNFα-treated HAECs with or without AMP-dNM treatment. (E) Histograms show mean densitometric readings ± SD of phosphorylated or non-phosphorylated proteins in insulin-stimulated cells normalized to loading control (β-ACTIN). All values were obtained from three independent experiments. (F) Immunocytochemical staining performed in TNFα-treated HAECs with or without AMP-dNM treatment. Representative images are shown (GM1, *green*; IRα, *red*; DAPI, *blue*; GM1 and IRα co-localization, *yellow*). ^*^P < 0.05. Control (Ctr): untreated cells.

In our previous report, we demonstrated that GM1 on the cell surface contributes to insulin resistance in HAECs [27]. We then hypothesized that increased GM1 levels in TNFα-treated HAECs result in impaired insulin signaling. We first examined the expression levels of insulin signaling molecules, such as the insulin receptor (IR) and IR substrate (IRS). IR expression was not altered at the cell surface after exposure to 1 ng/ml TNFα for 3 days (Figure 2A and Supplementary Figure 2A). Furthermore, mRNA levels of IRS1, IRS2 and eNOS did not significantly change (Figure 2B and Supplementary Figure 3A). Next, we examined insulin signaling in 1 ng/ml TNFα-treated HAECs with increased GM1 (Figure 2C) by monitoring the levels of phosphorylated protein kinase B (AKT) and eNOS, which are molecules involved in the insulin signaling cascade [29]. Western blot analysis showed that insulin-induced phosphorylation of AKT and eNOS was reduced in 1 ng/ml TNFα-treated HAECs compared to control cells without a concomitant significant reduction in AKT and eNOS levels (Figure 2D and 2E). In order to clarify the effect of increased GM1 levels, we used *N*-(5′-adamantane-1’-yl-methoxy)-pentyl-1-deoxynojirimycin (AMP-dNM). AMP-dNM is a specific inhibitor of glucosylceramide synthase that can be used to study the functional roles of endogenous gangliosides [25, 30]. In our previous study, we employed this inhibitor to investigate the effect of increased GM1 levels [27]. Treatment with AMP-dNM resulted in a decrease in GM1 levels of 1 ng/ml TNFα-treated HAECs that was comparable to the one detected in control cells (Figure 2C). Western blot analysis showed that reduction of insulin-induced phosphorylation of AKT and eNOS was not caused by AMP-dNM treatment, indicating that insulin signaling in TNFα-treated HAECs could normally occur upon AMP-dNM treatment (Figure 2D and 2E). In control HAECs, there were no significant differences in insulin signaling before and after AMP-dNM treatment, indicating that AMP-dNM does not affect insulin signaling (data not shown). It has been demonstrated that interaction between GM3/GM1 and IR is required for insulin resistance [27, 31]. We therefore examined whether GM1 and IR interacted in 1 ng/ml TNFα-treated HAECs. Immunocytochemical staining showed that GM1 co-localized with cell surface IR on 1 ng/ml TNFα-treated HAECs. Co-localization could not be observed upon AMP-dNM treatment (Figure 2F). These results indicate that increased levels of GM1 on the surface of 1 ng/ml TNFα-treated HAECs contribute to insulin resistance.

### Reduction of eNOS levels in long-term 1 ng/ml TNFα-treated HAECs is inhibited upon AMP-dNM treatment

**Figure 3:**
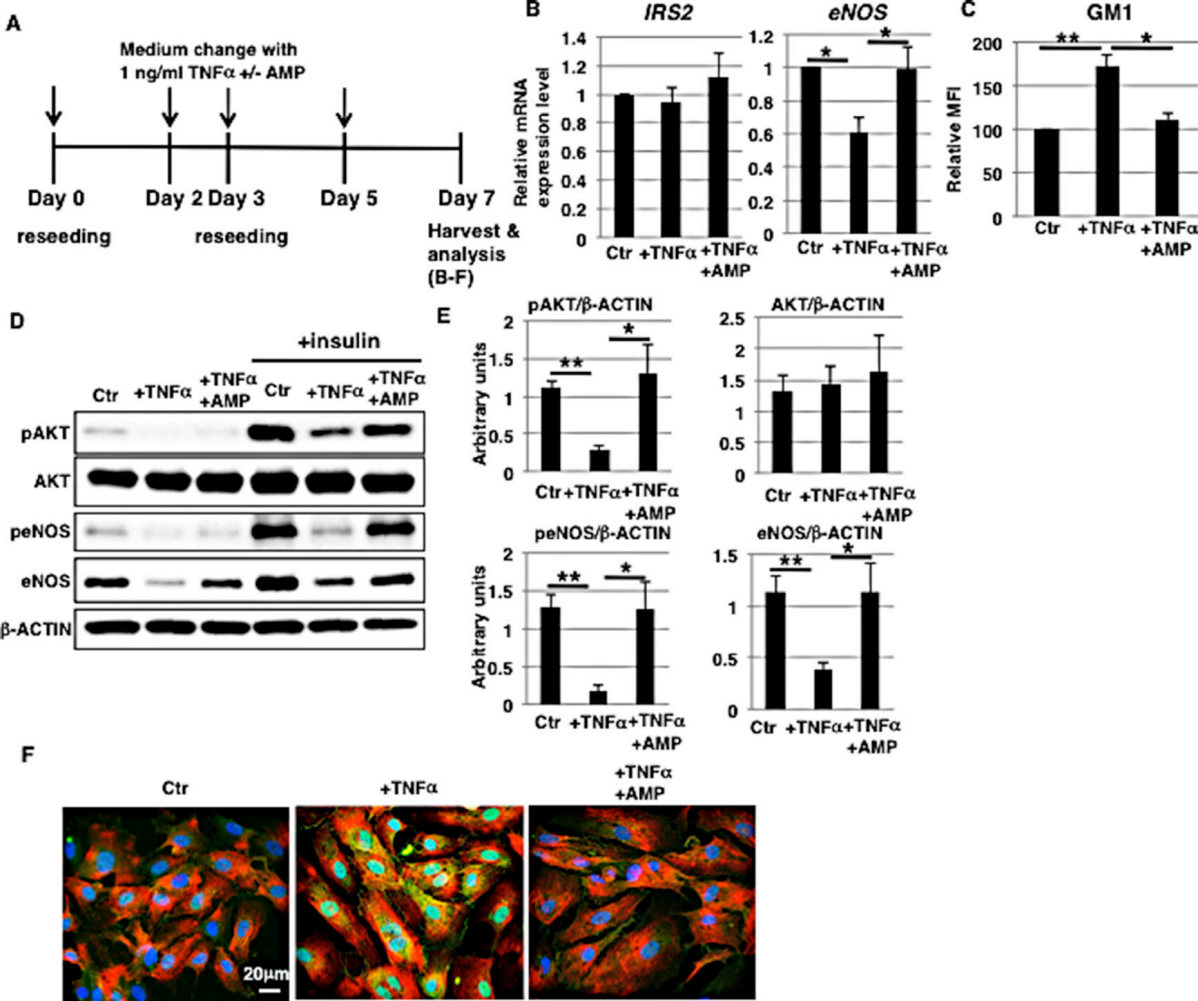
Reduction of eNOS levels in long-term 1 ng/ml TNFα-treated HAECs is inhibited by AMP-dNM treatment (**A**) At day 0, cells were reseeded with 1 ng/ml TNFα and with or without AMP-dNM in HAEC medium and incubated for 3 days. At day 3, cells were reseeded with 1 ng/ml TNFα and with or without AMP-dNM in HAEC medium and subsequently incubated for 4 days. At day 7, cells were harvested and analyzed (**B**–**F**). Control cells were grown in HAEC medium. (B) Real-time PCR analysis of *IRS2* and *eNOS* performed using cDNA derived from control and TNFα-treated HAECs with or without AMP-dNM treatment. Results shown were normalized against values obtained for control HAECs (value = 1). (C) Cell surface levels of GM1 in TNFα-treated HAECs with or without AMP-dNM treatment analyzed by flow cytometry. MFIs relative to control HAECs of three independent experiments are shown. (D) Western blot analysis of insulin signaling performed in TNFα-treated HAECs with or without AMP-dNM treatment. (E) Histograms show mean densitometric readings ± SD of phosphorylated or non-phosphorylated proteins in insulin-stimulated cells normalized to loading controls (β-ACTIN). All values were obtained from three independent experiments. (F) Immunocytochemical staining performed in TNFα-treated HAECs with or without AMP-dNM treatment. Representative images are shown (GM1, *green*; IRα, *red*; DAPI, *blue*; GM1 and IRα co-localization, *yellow*). ^*^P < 0.05; ^**^P < 0.01. Control (Ctr): untreated cells.

Chronic vascular inflammation leads to pathogenesis of vascular diseases, although the precise molecular mechanisms of vascular insulin resistance mediated by chronic inflammation are not well characterized [32, 33]. As *in vitro* model of chronic inflammation, we examined the long-term (7 days) effect of 1 ng/ml TNFα exposure on HAECs (Figure 3A). Cell surface IR expression was not significantly increased (Supplementary Figure 2B). Long-term (7 days) exposure significantly reduced mRNA levels of eNOS, but not of IRS2 (Figure 3B). Notably, AMP-dNM treatment inhibited downregulation of mRNA (Figure 3B) and protein levels of eNOS (Figure 3D and 3E) accompanying a reduction in GM1 levels (Figure 3C), suggesting that increased GM1 upon TNFα exposure may affect the expression of eNOS. Next, we examined insulin signaling in long-term 1 ng/ml TNFα-treated HAECs. Western blot analysis showed that insulin-induced phosphorylation of AKT and eNOS was reduced in long-term 1 ng/ml TNFα-treated HAECs compared to control cells, indicating that insulin signaling was impaired after long-term 1 ng/ml TNFα treatment (Figure 3D and 3E). In AMP-dNM treated cells, insulin-induced phosphorylation of AKT and eNOS was not reduced, indicating that the impairment of insulin signaling and down-stream eNOS activation (peNOS/β-ACTIN) in long-term 1 ng/ml TNFα-treated HAECs was not induced by AMP-dNM treatment (Figure 3D and 3E). Immunocytochemical staining showed that GM1 co-localized with cell surface IR in long-term 1 ng/ml TNFα-treated HAECs but not upon AMP-dNM treatment (Figure 3F), indicating that increased GM1 levels upon TNFα exposure contribute to impair insulin signaling. Thus, we demonstrated that long-term (7 days) exposure to 1 ng/ml TNFα induces GM1-dependent impairment of insulin signaling and down-stream eNOS activation together with a decrease in eNOS levels.

### GM1 expression is reversible in 1 ng/ml TNFα-treated HAECs

**Figure 4:**
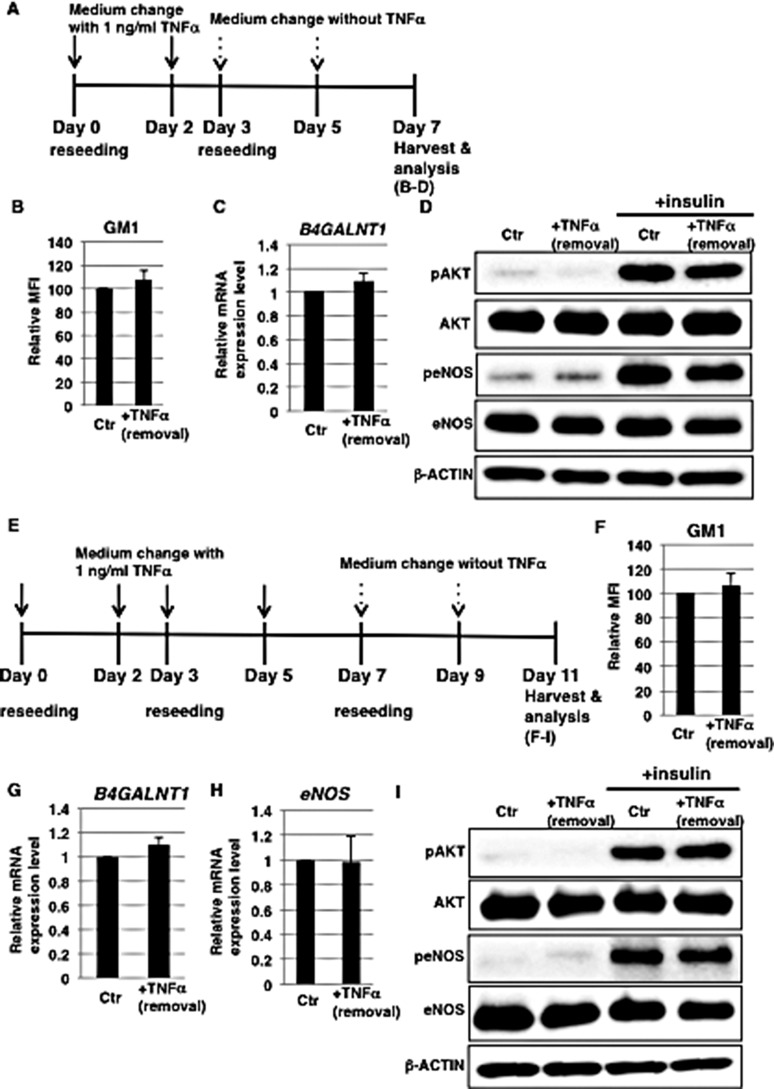
Induction of GM1 expression is reversible in 1 ng/ml TNFα-treated HAECs (**A**) At day 0, cells were reseeded with 1 ng/ml TNFα in HAEC medium and incubated for 3 days. At day 3, cells were reseeded without TNFα. Control cells were grown in HAEC medium. (**B**) Cell surface levels of GM1 in short-term (3 days) 1 ng/ml TNFα-treated HAECs 4 days after removal of TNFα analyzed by flow cytometry. MFIs relative to control HAECs of three independent experiments are shown. (**C**) Real-time PCR analysis of *B4GALNT1* performed using cDNA derived from control and short-term (3 days) 1 ng/ml TNFα-treated HAECs 4 days after removal of TNFα. Results shown were normalized against the values obtained for control HAECs (value = 1). Results are presented as means ± SD from three independent experiments. (**D**) Western blot analysis of insulin signaling performed for short-term (3 days) 1 ng/ml TNFα-treated HAECs 4 days after removal of TNFα. (**E**) At day 0, cells were reseeded with 1 ng/ml TNFα in HAEC medium and incubated for 3 days. At day 3, cells were reseeded with 1 ng/ml TNFα in HAEC medium and further incubated for 4 days. At day 7, cells were reseeded without TNFα. Control cells were grown in HAEC medium. (**F**) Cell surface levels of GM1 in long-term (7 days) 1 ng/ml TNFα-treated HAECs 4 days after removal of TNFα analyzed by flow cytometry. MFIs relative to control HAECs of three independent experiments are shown. (**G**) Real-time PCR analysis of *B4GALNT1* performed using cDNA derived from control and long-term (7 days) 1 ng/ml TNFα-treated HAECs 4 days after removal of TNFα. Results shown were normalized against values obtained for control HAECs (value = 1). Results are presented as means ± SD from three independent experiments. (**H**) Real-time PCR analysis of *eNOS* performed using cDNA derived from control and long-term (7 days) 1 ng/ml TNFα-treated HAECs 4 days after removal of TNFα. Results shown were normalized against the values obtained for control HAECs (value = 1). Results are presented as means ± SD from three independent experiments. (**I**) Western blot analysis of insulin signaling performed for long-term (7 days) 1 ng/ml TNFα-treated HAECs 4 days after removal of TNFα. Control (Ctr): untreated cells.

Prolonged inflammatory response has a detrimental effect on the function of vasculature, therefore appropriate control of the duration of an inflammatory response is important [34]. We speculated that a prolonged duration of vascular insulin resistance via increased GM1 expression may have a detrimental effect. In order to investigate the stability of TNFα-mediated GM1 induction, we treated HAECs with 1 ng/ml TNFα for 3 days and then removed TNFα (Figure 4A). The expression levels of GM1 and the enzyme that catalyzes its synthesis, *B4GALNT1*, were comparable to those of control cells upon removal of TNFα (Figure 4B and 4C) and insulin signaling was restored (Figure 4D). Furthermore, to determine the stability of GM1 and investigate the reduction of eNOS expression in long-term 1 ng/ml TNFα-treated HAECs, we examined their expression levels after removal of TNFα (Figure 4E). We found that the expression levels of GM1 and also *B4GALNT1* were comparable to those of control cells upon removal of TNFα (Figure 4F and 4G) and that insulin signaling and down-stream eNOS activation were restored together with eNOS levels (Figure 4H and 4I). Thus, we demonstrated that GM1 expression and insulin signaling are restored in 1 ng/ml TNFα-treated HAECs after removal of TNFα.

### eNOS activation is attenuated independently of GM1 in 10 ng/ml TNFα-treated HAECs

**Figure 5:**
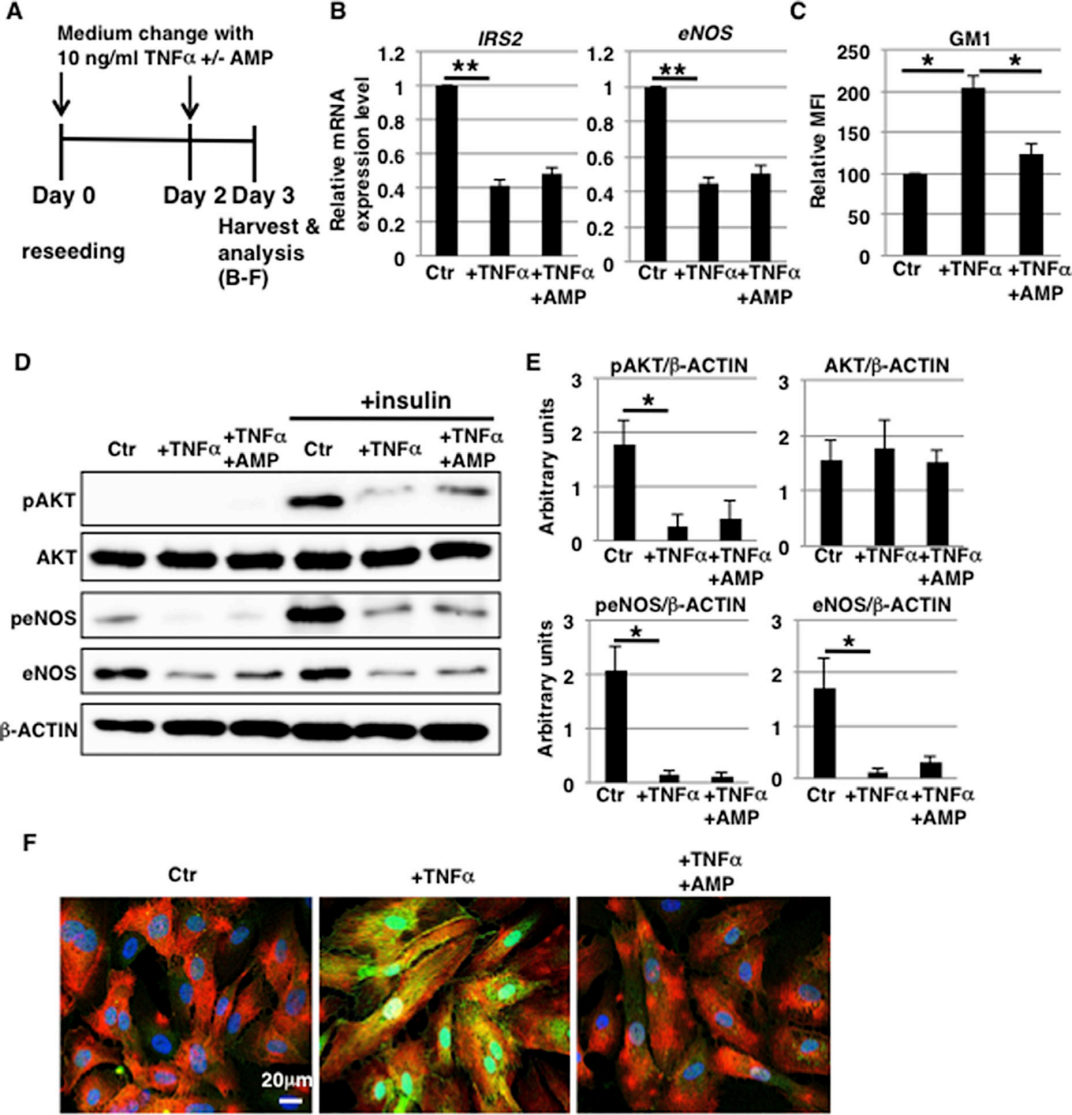
eNOS activation is attenuated independently of GM1 in 10 ng/ml TNFα-treated HAECs (**A**) At day 0, cells were reseeded with 10 ng/ml TNFα and with or without AMP-dNM in HAEC medium and incubated for 3 days. At day 3, cells were harvested and analyzed (**B**–**F**). Control cells were grown in HAEC medium. (B) Real-time PCR analysis of *IRS2* and *eNOS* performed using cDNA derived from control and TNFα-treated HAECs with or without AMP-dNM treatment. The results shown were normalized against the values obtained for control HAECs (value = 1). (C) Cell surface levels of GM1 in TNFα-treated HAECs with and without AMP-dNM treatment analyzed by flow cytometry. MFIs relative to control HAECs of three independent experiments are shown. (D) Western blot analysis of insulin signaling performed for TNFα-treated HAECs with or without AMP-dNM treatment. (E) Histograms show mean densitometric readings ± SD of phosphorylated or non-phosphorylated proteins in insulin-stimulated cells normalized to loading controls (β-ACTIN). All values were obtained from three independent experiments. (F) Immunocytochemical staining performed in TNFα-treated HAECs with or without AMP-dNM treatment. Representative images are shown (GM1, *green*; IRα, *red*; DAPI, *blue*; GM1 and IRα co-localization, *yellow*). ^*^P < 0.05; ^**^P < 0.01. Control (Ctr): untreated cells.

The intensity of inflammatory responses is an important factor in the pathogenesis of vascular diseases [33]. As described above, the increase in GM1 levels observed in TNFα-treated HAECs was dependent on TNFα concentration (above 1 ng/ml) (Figure 1A and 1B). We therefore examined the effect of highly concentrated TNFα exposure on HAECs (Figure 5A). Cell surface IR expression did not change upon short-term (3 days) exposure to 10 ng/ml TNFα (Supplementary Figure 2C). In contrast, compared with 1 ng/ml TNFα treatment, 10 ng/ml TNFα exposure for 3 days reduced mRNA levels of IRS2 and eNOS, but not of IRS1 (Figure 5B and Supplementary Figure 3B). Next, we examined insulin signaling in 10 ng/ml TNFα-treated HAECs as we did for 1 ng/ml TNFα-treated HAECs. Western blot analysis showed that insulin-induced phosphorylation of AKT and eNOS was reduced in 10 ng/ml TNFα-treated HAECs with a concomitant increase in GM1 levels compared to control cells, indicating a reduction in insulin signaling and down-stream eNOS activation (Figure 5C–5E). Treatment with AMP-dNM downregulated GM1 in 10 ng/ml TNFα-treated HAECs to levels comparable to the ones of control cells (Figure 5C). In addition, Western blot analysis showed that the impairment of insulin signaling and down-stream eNOS activation was not restored upon AMP-dNM treatment (Figure 5D and 5E), although immunocytochemical staining showed that GM1 did not co-localize with cell surface IR in 10 ng/ml TNFα-treated HAECs upon AMP-dNM treatment (Figure 5F). In 10 ng/ml TNFα-treated HAECs, downregulation of mRNA levels of IRS2 and of both mRNA and protein levels of eNOS was not inhibited upon AMP-dNM treatment, suggesting that these downregulation mechanisms observed after highly concentrated TNFα exposure are independent of GM1 (Figure 5B, 5D and 5E). Upon short-term (3 days) exposure to 10 ng/ml TNFα, HAECs acquired spindle-shaped fibroblast-like morphologies (Figure 1C). Real-time PCR, immunocytochemical staining and FACS analysis revealed that endothelial-mesenchymal transition (EndMT)-like differentiation was induced in 10 ng/ml TNFα-treated HAECs (Supplementary Figure 4). Taken together, these results indicate that insulin signaling and down-stream eNOS activation are attenuated independently of GM1 in 10 ng/ml TNFα-treated HAECs accompanying EndMT-like differentiation.

### Induction of GM1 expression is stable in 10 ng/ml TNFα-treated HAECs

**Figure 6:**
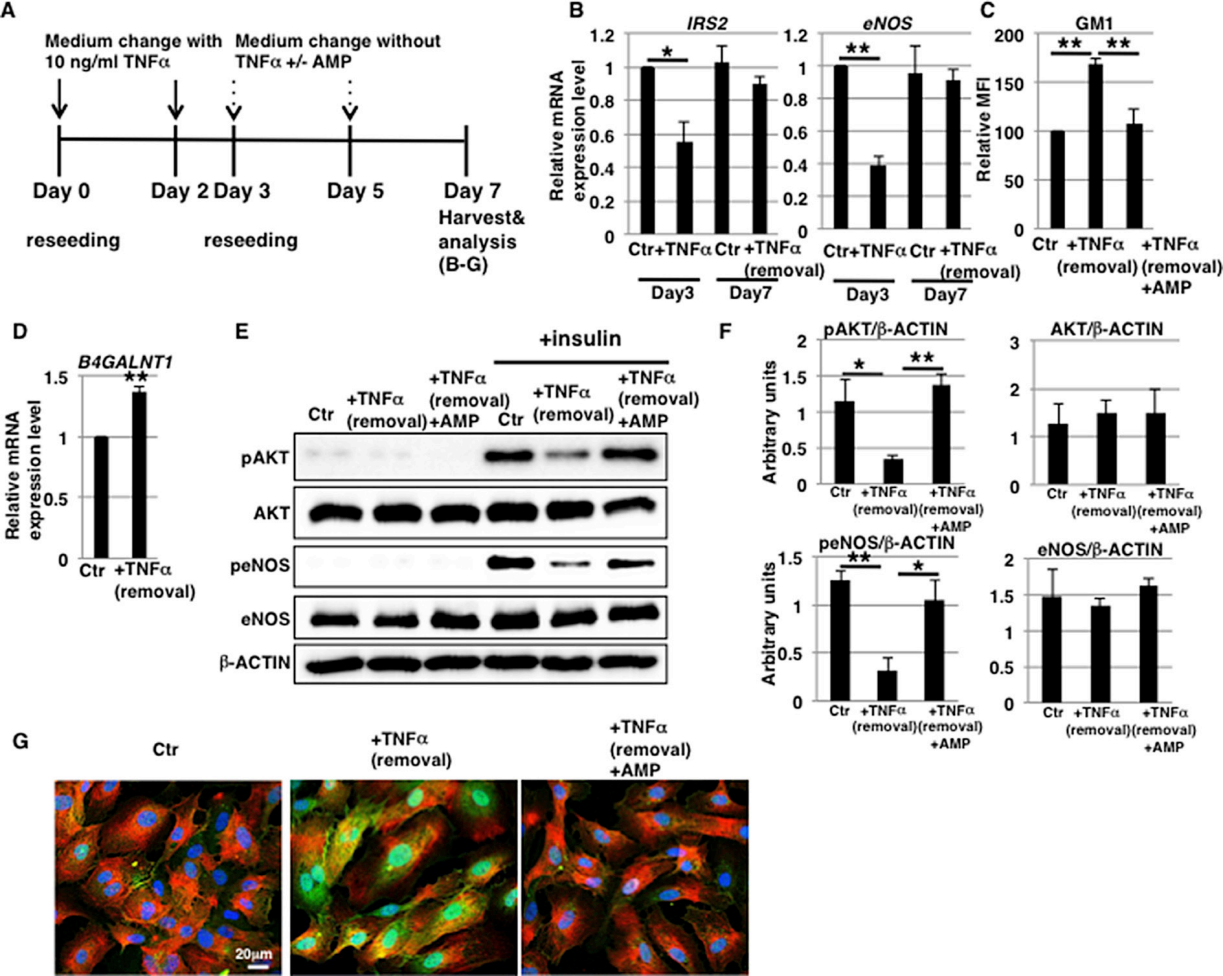
Induction of GM1 expression is stable in 10 ng/ml TNFα-treated HAECs (**A**) At day 0, cells were reseeded with 10 ng/ml TNFα in HAEC medium and incubated for 3 days. At day 3, cells were reseeded without TNFα and with or without AMP-dNM in HAEC medium and further incubated for 4 days. At day 7, cells were harvested and analyzed (B-G). Control cells were grown in HAEC medium. (**B**) Real-time PCR analysis of *IRS2* and *eNOS* performed using cDNA derived from control, 3-day TNFα-treated HAECs and 3-day TNFα-treated HAECs 4 days after removal of TNFα. Results shown were normalized against values obtained for control HAECs (value = 1). (**C**) FACS analysis of cell surface GM1 performed in control and TNFα-treated HAECs with or without AMP-dNM treatment for 4 days after removal of TNFα. MFIs relative to control HAECs are shown. (**D**) Real-time PCR analysis of *B4GALNT1* performed using cDNA derived from control and TNFα-treated HAECs 4 days after removal of TNFα. Results shown were normalized against values obtained for control HAECs (value = 1). (**E**) Western blot analysis of insulin signaling performed for TNFα-treated HAECs with or without AMP-dNM treatment 4 days after removal of TNFα. (**F**) Histograms show mean densitometric readings ± SD of phosphorylated or non-phosphorylated proteins in insulin-stimulated cells normalized to loading controls (β-ACTIN). All values were obtained from three independent experiments. (**G**) Immunocytochemical staining performed in TNFα-treated HAECs with or without AMP-dNM treatment 4 days after removal of TNFα. Representative images are shown (GM1, *green*; IRα, *red*; DAPI, *blue*; GM1 and IRα co-localization, *yellow*). ^*^P < 0.05; ^**^P < 0.01. Control (Ctr): untreated cells.

After short-term (3 days) 10 ng/ml TNFα treatment, the endothelial marker CD31 was fully expressed in HAECs (Supplementary Figure 4C). It could therefore be hypothesized that EndMT-like differentiation was partial and could be reversed. We tested this in short-term 10 ng/ml TNFα-treated HAECs (Figure 6A). Four days after withdrawal of TNFα, mRNA levels of IRS2 and eNOS in short-term 10 ng/ml TNFα-treated HAECs were comparable to those of control cells (Figure 6B). This restoration accompanied an almost complete reinstatement of the expression levels of EndMT markers (Supplementary Figure 5). These results suggest that downregulation of mRNA levels of IRS2 and eNOS in 10 ng/ml TNFα-treated HAECs is dependent on EndMT-like differentiation. In summary, we showed that EndMT-like differentiation in short-term 10 ng/ml TNFα-treated HAECs was reversible, whereas GM1 and *B4GALNT1* expression remained at high levels (Figure 6C and 6D), unlike for 1 ng/ml TNFα-treated HAECs.

It has been recently reported that long-term (6 days) exposure to highly concentrated TNFα induces premature senescence in human umbilical vein ECs via reactive oxygen species (ROS) production [35]. In our previous report, we showed that premature senescence induced by ROS increases GM1 levels with a concomitant increased expression of *B4GALNT1* in HAECs [27]. We therefore examined the possible induction of premature senescence in HAECs reversed from EndMT-like differentiation. In our senescence assay (Supplementary Figure 6), the senescence marker *p16*^*INK4a*^ and senescence-associated β-galactosidase (SA-β-Gal) levels were moderately but consistently increased in reversed HAECs compared to control cells, suggesting that cellular senescence was partially induced in reversed HAECs. Induction of GM1 upon 10 ng/ml TNFα exposure for 3 days therefore appeared to be highly stable, presumably depending on partial induction of premature senescence, even in the absence of initiating stimuli.

It is likely that GM1-dependent insulin resistance is still induced in reversed HAECs with increased GM1 levels. We next examined insulin signaling in reversed HAECs. Western blot analysis showed that insulin-induced phosphorylation of AKT and eNOS was reduced in reversed HAECs compared to control cells, without a concomitant reduction of AKT, eNOS and cell surface IR expression (Figure 6E, 6F and Supplementary Figure 2D). Treatment with AMP-dNM reduced GM1 levels in reversed HAECs to the same extent as in control cells (Figure 6C). We did not detect a reduction of insulin-induced phosphorylation of AKT and eNOS in AMP-dNM treated cells, indicating that the impairment of insulin signaling was not induced by AMP-dNM treatment (Figure 6E and 6F). Immunocytochemical staining showed that GM1 co-localized with cell surface IR in reversed HAECs but not upon AMP-dNM treatment (Figure 6G), indicating that the residual GM1 in reversed HAECs contributes to impair insulin signaling. These results clearly indicate that the residual GM1 in reversed HAECs is responsible for insulin resistance. Thus, we demonstrated that short-term exposure to 10 ng/ml TNFα induces reversible downregulation of IRS2 and eNOS due to EndMT-like differentiation and it is also likely to induce irreversible GM1 expression, probably resulting in chronic insulin resistance.

### Aging and senescence affect eNOS levels due to increased GM1 in short-term 1 ng/ml TNFα-treated HAECs

**Figure 7:**
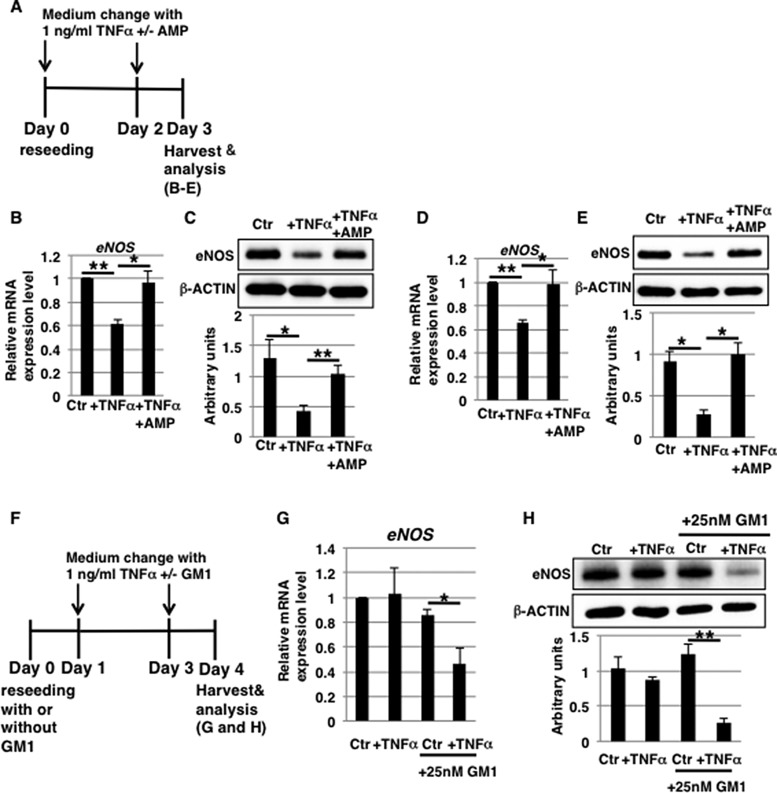
Aging and senescence affect eNOS levels due to increased GM1 levels in short-term 1 ng/ml TNFα-treated HAECs (**A**) At day 0, aged/senescent HAECs were reseeded with 1 ng/ml TNFα in HAEC medium with or without AMP-dNM and incubated for 3 days. At day 3, cells were harvested and analyzed (**B**–**E**). (**F**) At day 0, non-aged HAECs were reseeded with or without 25 nM GM1 in HAEC medium. At day 1, cells were treated with 1 ng/ml TNFα with or without 25 nM GM1 in HAEC medium and incubated for 3 days. At day 4, cells were harvested and analyzed (**G** and **H**). Control cells were grown in HAEC medium. (B, D and G) Real-time PCR analysis of *eNOS* performed using cDNA derived from short-term (3 days) 1 ng/ml TNFα-treated senescent HAECs with or without AMP-dNM treatment (B), short-term (3 days) 1 ng/ml TNFα-treated aged HAECs with or without AMP-dNM treatment (D), and short-term (3 days) 1 ng/ml TNFα-treated GM1-overexpressing HAECs (G). Results shown were normalized against values obtained for control HAECs (value = 1). (C, E and H) Western blot analysis of eNOS performed for short-term (3 days) 1 ng/ml TNFα-treated senescent HAECs with or without AMP-dNM treatment (C), short-term (3 days) 1 ng/ml TNFα-treated aged HAECs with or without AMP-dNM treatment (E), and short-term (3 days) 1 ng/ml TNFα-treated GM1-overexpressing HAECs (H). Histograms below Western blot panels show mean eNOS densitometric readings ± SD normalized to those of the loading controls (β-ACTIN). All values were obtained from three independent experiments. ^*^P < 0.05; ^**^P < 0.01. Control (Ctr): untreated cells.

In humans, aging and senescence affect cellular responses, leading to pathological disorders [36]. As shown in Figure 3B–3E, increased levels of GM1 in cell membranes affect the expression of eNOS upon TNFα exposure. We showed in our previous report that GM1 levels are increased in aged and senescent HAECs [27]. In order to clarify the effect of aging and senescence on the response of ECs to TNFα *in vivo*, we examined whether short-term (3 days) exposure to 1 ng/ml TNFα reduced eNOS levels in aged/senescent HAECs with increased GM1 levels (Figure 7A). We found that even short-term exposure to 1 ng/ml TNFα reduced mRNA and protein levels of eNOS in senescent as well as aged HAECs (Figure 7B–7E). This reduction was not induced by AMP-dNM treatment (Figure 7B–7E), which downregulates GM1 expression in aged/senescent HAECs (Supplementary Figure 7A–7C), suggesting that increased GM1 levels in aged/senescent HAECs affect the expression of eNOS after short-term (3 days) exposure to 1 ng/ml TNFα. Western blot analysis showed that insulin signaling was reduced both in control and TNFα-treated aged HAECs and this reduction was not restored upon AMP-dNM treatment (Supplementary Figure 7D). As suggested in our previous report, a partial reduction of GM1 levels in aged HAECs upon AMP-dNM treatment is likely to be one of the reasons why restoration of insulin signaling in aged HAECs could not be observed upon AMP-dNM treatment [27]. Furthermore, to confirm that increased GM1 levels contribute to downregulate eNOS, we investigated eNOS levels in GM1-overexpressing HAECs, which are produced from non-aged HAECs incubated with exogenous GM1 (Figure 7F). In GM1-overexpressing HAECs (Supplementary Figure 7E), short-term (3 days) exposure to 1 ng/ml TNFα resulted in reduced eNOS levels (Figure 7G and 7H), clearly indicating that increased GM1 levels are associated with lower eNOS levels. Thus, we demonstrated that even short-term exposure to 1 ng/ml TNFα induces downregulation of eNOS levels in aged/senescent HAECs due to increased GM1 levels, suggesting that even low concentrations of circulating TNFα represent a risk factor for vascular insulin resistance caused by reduced eNOS levels in elderly people.

## DISCUSSION

Inflammation has a detrimental effect on the function of vasculature when the mechanisms that regulate the intensity and duration of an acute inflammatory response are compromised. For example, an excessive inflammatory response during sepsis results in organ failure and death due to profound and systemic increases in endothelial cell permeability, while chronic vascular inflammation leads to pathogenesis of type 2 diabetes and drives progression of atherosclerosis [32, 33, 37]. Here, we show the contribution of GM1 to vascular insulin resistance as a vascular inflammatory response to TNFα stimulation. Our results suggest that GM1 is a key player in the regulation of the intensity and duration of insulin signaling. GM1 contributes to signal regulation at the cell surface or to the regulation of eNOS expression during TNFα response. It is likely that chronic vascular insulin resistance leading to initiation and progression of vascular diseases is dependent on extracellular GM1 expression and therefore GM1 is a good candidate extracellular target for prevention and cure of vascular diseases.

As shown in Figure 1F, GM1 is synthesized by glycosyltransferases and sialidase (*NEU3*). Altered expression of these enzymes leads to an increased production of GM1. In fact, overexpression of *B4GALNT1*, *B3GALT4* or *NEU3* in mammalian cells was reported to induce an increase in the levels of GM1 [20, 28]. In this study, we found that expression of *B4GALNT1*, which catalyzes the synthesis of GM2 (a GM1 precursor), was upregulated in TNFα-treated HAECs. Furthermore, we observed a reduced expression of *ST8SIA1*, which is not part of the GM2 and GM1 synthetic pathway, in TNFα-treated HAECs. It can be therefore suggested that the GM2 and GM1 synthetic pathway is predominant in TNFα-treated HAECs. In the GM1 synthetic pathway, expression of *B3GALT4,* which catalyzes the synthesis of GM1, was not significantly altered compared with *B4GALNT1*. We speculate that expression levels of *B3GALT4* may be adequate for the conversion of GM2 into GM1 in TNFα-treated HAECs. We observed a decrease in the levels of GD1a, but not of *ST3GAL2* and *NEU3* (which modulates the expression of GD1a) in TNFα-treated HAECs. Conversion of GM1 to GD1a may therefore be a rate-limiting step in HAECs. Thus, these results provide a possible explanation for the increased levels of GM1 in TNFα-treated HAECs. Further research is required to clarify the mechanisms regulating the expression of these enzymes and to identify the key regulators of GM1 in TNFα-treated HAECs.

The effect of increased GM1 levels on other functions of ECs is currently unknown, but, as shown here, stable GM1 expression after removal of highly concentrated TNFα may contribute to other chronic dysfunctions of ECs. Further studies are required to clarify the contribution of GM1 to other functions of ECs, but we propose that GM1 is an interesting target for prevention of vascular diseases caused by chronic vascular dysfunctions. TNFα levels are increased after acute myocardial infarction and induce acute inflammatory responses, including vascular insulin resistance. After acute myocardial infarction, dysregulation of immune pathways, impaired suppression of post-infarction inflammation, and perturbed spatial containment of the inflammatory response may cause adverse remodeling in patients contributing to heart failure [38]. Based on our results, we speculate that chronic vascular dysfunctions caused by residual GM1 after acute myocardial infarction may be involved in the initiation and development of heart failure.

eNOS expression upon highly concentrated (above 10 ng/ml) TNFα exposure is regulated through NF-κB, *eNOS* 3′-UTR binding proteins, and miR-155 [39–41]. In this study, we showed that long- and short-term exposure to 1 ng/ml TNFα induce a reduction of eNOS levels in non-senescent HAECs and in aged/senescent HAECs, respectively (Figure 3B–3E and 7). In these stimulated cells, increased GM1 levels contributed to eNOS downregulation presumably driving changes in the intensity of TNFα signaling. The TNF-receptor is controversially known to be functional on lipid rafts or non-raft regions [42, 43]. Increased GM1 levels affect cell surface expression of raft-associated proteins and contribute to reduce membrane fluidity [19–21]. Thus, it is possible that increased GM1 levels affect the cell surface distribution of the TNF-receptor leading to changes in signal transduction. We therefore speculate that long- and short-term exposure to low concentrations of TNFα induce downregulation of eNOS levels in non-senescent HAECs and in aged/senescent HAECs, respectively, via activation of *eNOS* 3′-UTR binding proteins and miR-155 depending on the intensity of TNFα/NF-κB signaling. These effects are similar to the ones induced by highly concentrated (above 10 ng/ml) TNFα exposure. Further studies, however, are required to clarify whether increased GM1 levels affect the localization and function of the TNF-receptor and the intensity of TNFα/NF-κB signaling.

We have shown here that increased GM1 levels in aged and senescent ECs contribute to vascular insulin resistance caused by reduced eNOS levels even upon exposure to low concentrations of TNFα. Notably, serum TNFα levels are known to be increased with aging and are associated with age-related diseases such as periodontal disease [12, 44]. Increased GM1 levels as a consequence of aging and senescence may contribute to the high risk of vascular diseases caused by vascular insulin resistance via reduced eNOS levels in elderly people. Our findings therefore suggest that GM1 is an attractive target for the detection, prevention, and treatment of insulin resistance, particularly in elderly people. At the moment, beraprost sodium is available as a potential therapeutic for vascular insulin resistance that acts by upregulating eNOS levels, but therapeutic strategies still need further development [9]. Inhibition of GM1 synthesis (via AMP-dNM treatment and targeting *B4GALNT1*) and of the interaction between GM1 and IR or TNFα signaling molecules could be employed to develop new extracellular therapeutic strategies. For the clinical translation of these findings free from side effects, further studies are required to identify the key regulators of GM1 homeostasis and to clarify the molecular mechanisms underlying insulin resistance and modulation of TNFα signaling intensity via GM1.

In conclusion, our data demonstrate that GM1 levels on the cell membrane of HAECs change depending on the time of exposure to TNFα and its concentration, and that these fluctuations are associated with specific extracellular/intracellular regulation of the insulin signaling cascade. Furthermore, we have demonstrated that aging and senescence affect the regulation of insulin resistance depending on GM1. Thus, based on our experiments mimicking *in vivo* acute or chronic inflammatory situations, we propose that GM1 is an attractive target for prevention and cure of vascular diseases including aging-related inflammatory diseases.

## MATERIALS AND METHODS

### Cell culture

HAECs from a 27-year-old male and an 81-year-old male were purchased from a commercial vendor (Lonza, Walkersville, MD, USA). Experiments were mainly performed using HAECs obtained from a 27-year-old male. Cells were grown in endothelial growth medium-2 (EGM-2) supplemented with growth factors, antibiotics, and 2% fetal bovine serum (FBS) (EGM-2 SingleQuot; Lonza). Cells were passaged at 80% confluence and seeded at a density of 2,500–5,000 cells/cm^2^. All experiments were performed at a confluence of 75–80%. Population doubling levels (PDLs) were calculated at each passage using the following equation: *n* = (log2X – log2Y), where *n* = PDL, X = number of cells at end of passage, and Y = number of seeded cells at beginning of passage. The PDL at first plating of a newly purchased cell stock was defined as PDL 0. In this study, cells (HAECs of a 27-year-old male, non-aged) with a PDL of 14–17 and cells (HAECs of an 81-year-old male, aged) with a PDL of 7–11 were used as early passage (EP, non-senescent) cells, and cells (HAECs of a 27-year-old male) with a PDL of 29–30 were used as senescent (SEN) cells. For reduction of GM1, HAECs were treated with either vehicle (ethanol) only or 10 µM AMP-dNM (Cayman Chemical, Ann Arbor, MI, USA) throughout the entire culture period until they were used for experiments. For overexpression of GM1, HAECs were cultured with either vehicle (methanol) only or 25 nM GM1 (Sigma-Aldrich, St. Louis, MO, USA) in HAEC culture medium for 24 h.

### SA-β-Gal assay

SA-β-Gal activity was assayed by using a senescence detection kit (BioVision Inc., Milpitas, CA, USA) as described in our previous reports [27, 45].

### FACS analysis

FACS analysis was performed as described in our previous reports [22, 39]. Briefly, cells were harvested with the Accutase^®^ cell detachment solution (Merck Millipore, Billerica, MA, USA) and dissociated single cells were incubated with primary antibodies or fluorescein isothiocyanate (FITC)-conjugated antibodies diluted in FACS buffer (0.5% [w/v] bovine serum albumin [BSA] and 0.1% [w/v] sodium azide in phosphate-buffered saline [PBS]) for 30 min on ice. After washing, the cell suspension was incubated with Alexa Fluor^®^ 488-conjugated secondary antibodies (Molecular Probes, Eugene, OR, USA) diluted in FACS buffer for 30 min on ice. For detection of GM1, cells were incubated with Alexa Fluor^®^ 647-conjugated cholera toxin B subunit (Molecular Probes) diluted in FACS buffer for 30 min on ice. Cell sorting and analysis were performed using a FACSAria™ Cell Sorter (Becton Dickinson, Franklin Lakes, NJ, USA). We used the following FITC-conjugated antibodies and primary antibodies: FITC-conjugated anti-CD31 (Becton Dickinson), FITC-conjugated anti-CD10 (Becton Dickinson), FITC-conjugated anti-CD44 (Becton Dickinson), FITC-conjugated anti-CD90 (Miltenyi Biotec Inc., Auburn, CA, USA), anti-STRO-1 (R&D Systems Inc., Minneapolis, MN, USA), anti-N-CADHERIN (Sino Biological Inc., Beijing, China), anti-GM3 (NBT Laboratories Inc., Tokyo, Japan), anti-monosyalotrihexosylceramide (GM2) (TCI, Tokyo, Japan), anti-monosyalotrihexosylceramide (GD1a) (TCI), anti-ganglioside GD3 (Merck Millipore), anti-ganglioside GD2 (TCI), anti-ganglioside GD1b (TCI), and anti-IRα (Abcam, Cambridge, UK). Mean fluorescence intensities (MFIs) were calculated by subtracting the intensities of the controls.

### Immunoblotting

To investigate insulin signaling, the cell culture medium was replaced with EGM-2 containing 1% (v/v) FBS for 6 h and cells were stimulated for 5 min with 1 µM human insulin (Wako, Osaka, Japan). Cells were lysed with lysis buffer (50 mM Tris-HCl pH 7.4, 150 mM NaCl, and 1% [v/v] Triton™ X-100) containing protease and phosphatase inhibitor cocktails (Roche, Indianapolis, IN, USA). Samples prepared as described above were resolved by SDS-PAGE and subsequently transferred onto PVDF membranes (Merck Millipore). After blocking, membranes were incubated with the following primary antibodies: monoclonal rabbit anti-AKT (4691; Cell Signaling Technology, Danvers, MA, USA), monoclonal rabbit anti-phosphorylated AKT (pAKT) (Ser473; 4060; Cell Signaling Technology), monoclonal mouse anti-eNOS (610297; Becton Dickinson), monoclonal rabbit anti-phosphorylated eNOS (peNOS) (Ser1177; 9570; Cell Signaling Technology), and monoclonal mouse anti-β-ACTIN (A5316; Sigma-Aldrich). Membranes were subsequently incubated with the appropriate peroxidase-conjugated secondary antibodies (Cell Signaling Technology), washed and developed with ECL™ Prime reagents (GE Healthcare, Piscataway, NJ, USA). The signal was detected using a GE Healthcare ImageQuant LAS3000m.

### Immunostaining

Cells were fixed with 4% (w/v) paraformaldehyde and washed. Subsequently, cells were permeabilized with 0.2% (v/v) Triton^™^ X-100 and blocked with PBS containing 1% (w/v) BSA and 5% (v/v) normal goat serum. After washing, cells were incubated with anti-FSP1 (ab27957; Abcam) at 4°C overnight. After washing, cells were stained with an Alexa Fluor^®^ 546-conjugated secondary antibody (Molecular Probes) and then counterstained with DAPI. Immunofluorescence images were acquired using a fluorescence microscope (Leica Microsystems, Wetzlar, Germany). For analysis of gangliosides with or without IRα, cells were stained without being previously permeabilized. Immunofluorescence images were acquired using a confocal laser scanning microscope (Leica Microsystems).

### Real-time PCR

Total RNA was isolated from cells using the RNeasy plus mini kit (QIAGEN, Hilden, Germany) and subsequently reverse-transcribed using the ReverTra Ace^®^ qPCR RT Kit (Toyobo, Osaka, Japan). Real-time PCR was performed using the Power Sybr^®^ Green kit (Applied Biosystems, Foster City, CA, USA) and the StepOnePlus™ real-time PCR system (Applied Biosystems). Primer sets for real-time PCR are listed in Table 1.

**Table 1:** List of primer sets for real-time PCR

Gene	Forward primer	Reverse primer
*ST3GAL5*	AGGAATGTCGTCCCAAGTTTG	GGAGTAAGTCCACGCTATACCT
*B4GALNT1*	ACAGCAGACACAGTCCGGTTCT	GCGGGTGTCTTATGCGGATA
*ST8SIA1*	TACTCTCTCTTCCCACAGG	GACAAAGGAGGGAGATTGC
*B3GALT4*	GAAGGAGGCCAGGTTTTGC	CCCGGCCCAAGTACAGAAG
*ST3GAL2*	TGGACGGGCACAACTTCA	TGCCAACATCCTGCTCAAAG
*NEU3*	AATGTGAAGTGGCAGAGGTGA	TCACAGAGCTGTCGACTCAGG
*IRS2*	GCAGAACATCCACGAGACCAT	GGAACTCGAAGAGCTCCTTGAG
*FSP1*	TGGAGAAGGCCCTGGATGT	CCCTCTTTGCCCGAGTACTTG
*CD44*	ACCTGCCCAATGCCTTTG	GACATAGCGGGTGCCATCAC
*SNAIL*	CCCCAATCGGAAGCCTAACT	GCTGGAAGGTAAACTCTGGATTAGA
a*SMA*	CACCATCGGAAATGAACGTTT	GACTCCATCCCGATGAAGGA
*SM22*	GGCGTGATTCTGAGCAAGCT	CACCTTCACCGGCTTGGA
*N-CADHERIN*	TGGGAATCCGACGAATGG	GCAGATCGGACCGGATACTG
*vWF*	CGGCTTGCACCATTCAGCTA	TGCAGAAGTGAGTATCACAGCCATC
*eNOS*	CGGCATCACCAGGAAGAAGA	TGAGCGAGGCGGAGATCT
*p16*	CCAACGCACCGAATAGTTACG	GGGCGCTGCCCATCA
*p21*	TGGAGACTCTCAGGGTCGAAA	GCGTTTGGAGTGGTAGAAATCTG
*p53*	TCTCCCCAGCCAAAGAAGAA	CCACGGATCTGAAGGGTGAA
*β-ACTI*N	GGTCATCACCATTGGCAATGAG	TACAGGTCTTTGCGGATGTCC

### Statistical analysis

Western blot images were densitometrically analyzed with the ImageJ software (National Institute of Health). Values were expressed as means ± SD from at least three independent experiments. Student’s t tests for independent samples were used for statistical analysis.

## SUPPLEMENTARY MATERIALS FIGURES



## Abbreviations

AMP-dNM: *N*-(5′-adamantane-1′-yl-methoxy)-pentyl-1-deoxynojirimycin
BSA: bovine serum albumin
ECs: endothelial cells
EndMT: endothelial-mesenchymal transition
eNOS: endothelial NO synthase
GM3: monosialodihexosylganglioside
HAEC: human aortic endothelial cell
IR: insulin receptor
IRS: IR substrate
MFI: mean fluorescent intensity
PBS: phosphate-buffered saline
ROS: reactive oxygen species
TNFα: tumor necrosis factor-alpha
