# Supplementary material for: Ganglioside GM1 contributes to extracellular/intracellular regulation of insulin resistance, impairment of insulin signaling and down-stream eNOS activation, in human aortic endothelial cells after short- or long-term exposure to TNFα
Source: Oncotarget. 2017 Dec 15;9(5):5562–77. doi: 10.18632/oncotarget.23726 (PMC5814158; doi:10.18632/oncotarget.23726)
Supplement: Supplementary file 1 [file oncotarget-09-5562-s001.pdf]

Ganglioside GM1 contributes to extracellular/intracellular regulation of insulin resistance, impairment of insulin signaling and down-stream eNOS activation, in human aortic endothelial cells after short- or long-term exposure to TNFα

SUPPLEMENTARY MATERIALS

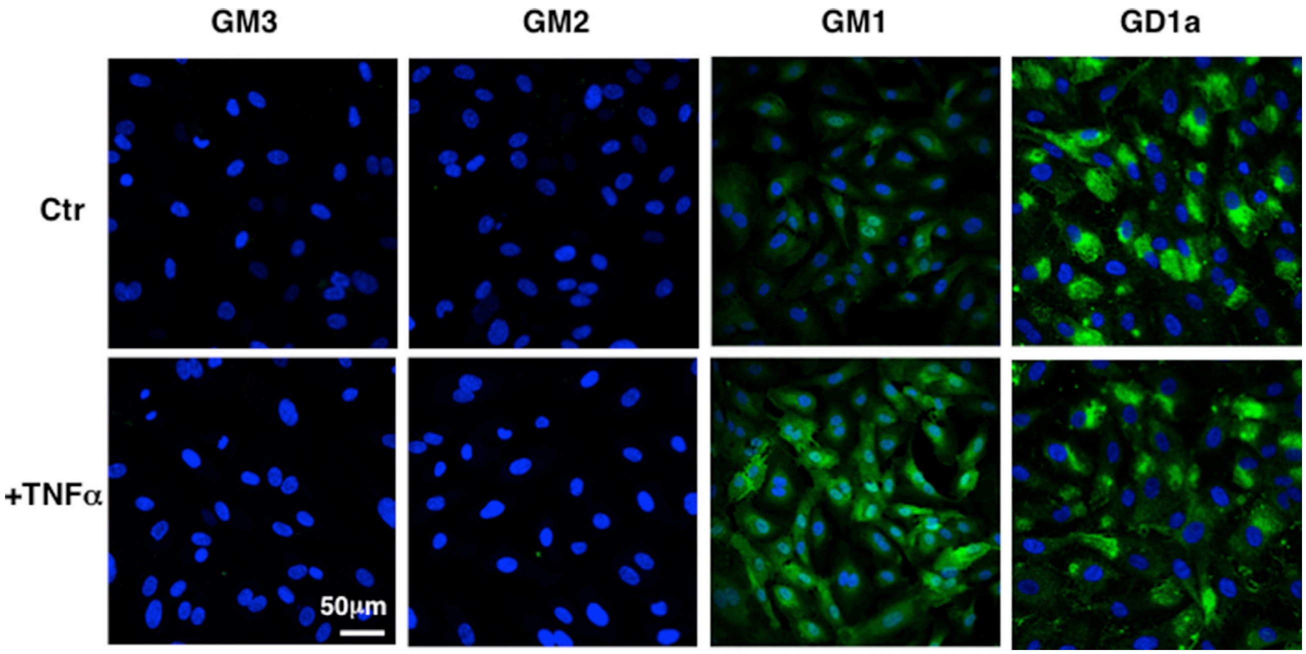

Supplementary Figure 1: Expression levels of gangliosides. Immunocytochemical staining of gangliosides (GM3, GM2, GM1 and GD1a) performed in short-term (3 days) 1 ng/ml TNFα-treated HAECs. Representative images are shown (gangliosides, *green*; DAPI, *blue*). Control (Ctr): untreated cells.

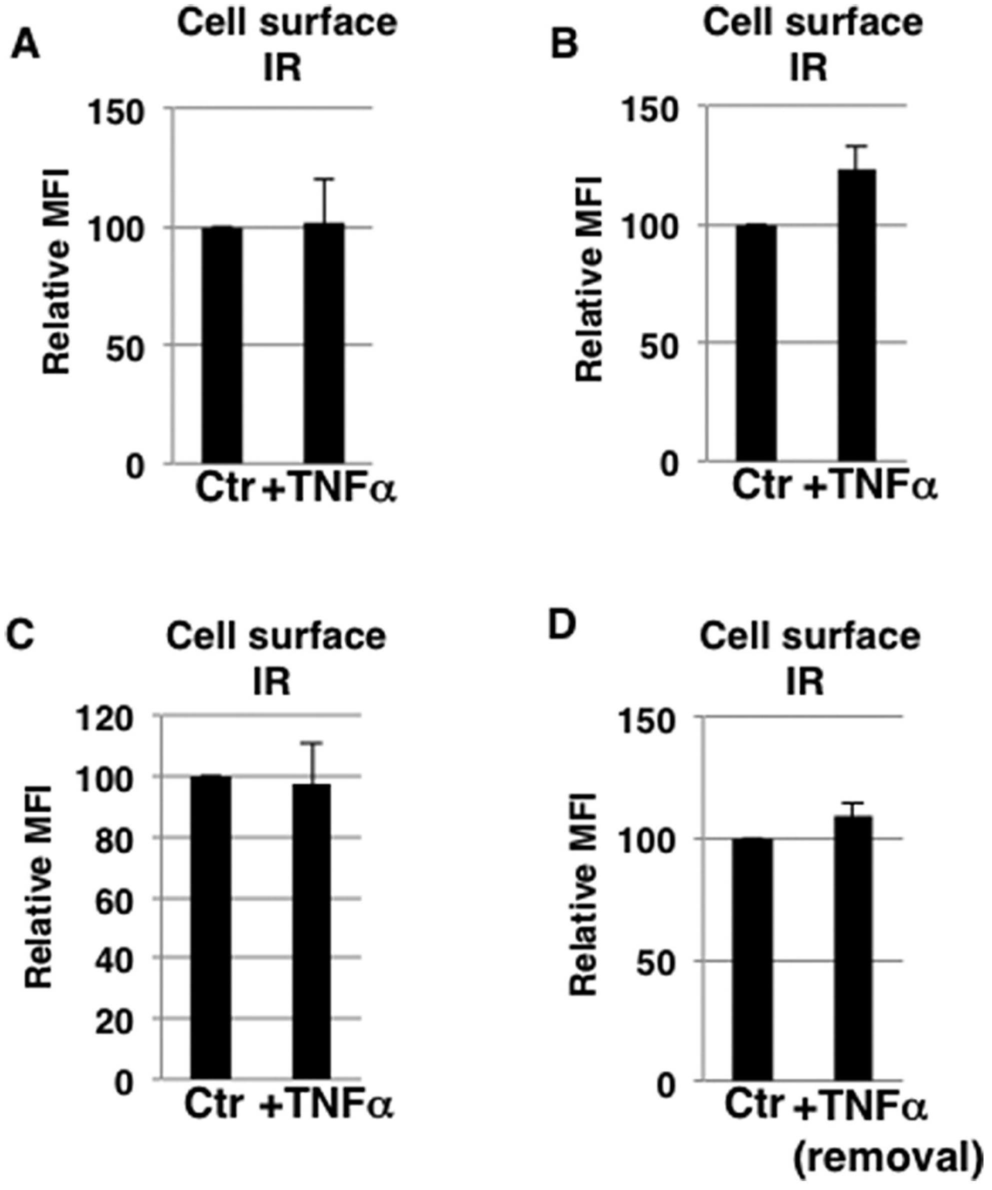

Supplementary Figure 2: Expression levels of cell surface IRα do not change upon TNFα exposure. FACS analysis for cell surface IRα of control and TNFα-treated HAECs [(**A**) 1 ng/ml TNFα (3 days), (**B**) 1 ng/ml TNFα (7 days), (**C**) 10 ng/ml TNFα (3 days), (**D**) 10 ng/ml TNFα (3-day treatment and 4 days after removal)]. MFIs relative to control HAECs are shown. Results are shown after normalization to the values obtained for control HAECs. Results are presented as means ± standard deviation (SD) from three independent experiments. Control (Ctr): untreated cells.

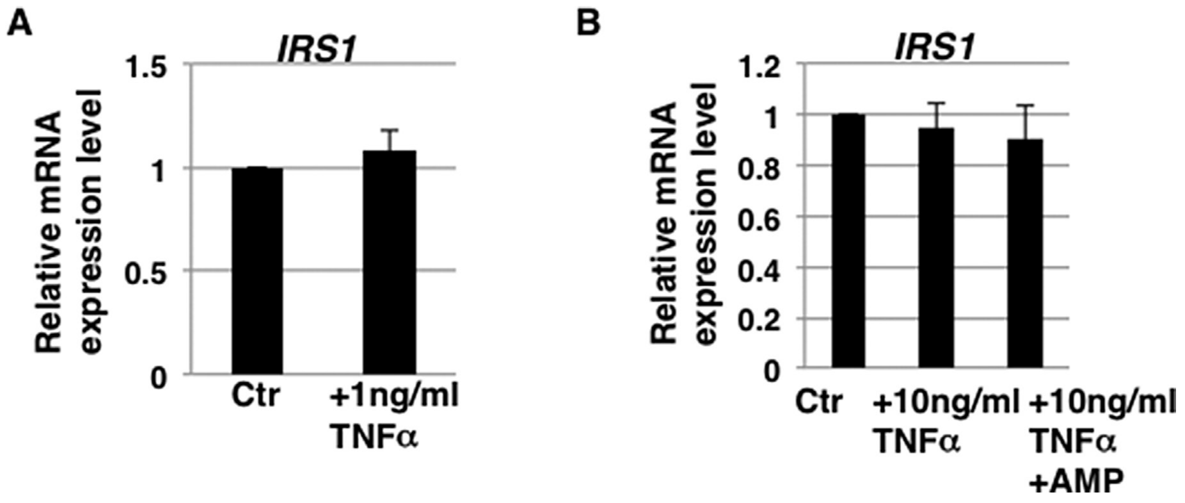

Supplementary Figure 3: Expression levels of IRS1 do not change upon TNFα exposure. Real-time PCR analysis of *IRS1* performed using cDNA derived from short-term (3 days) 1 ng/ml TNFα-treated HAECs (A) or short-term (3 days) 10 ng/ml TNFα-treated HAECs with or without AMP-dNM treatment (B). Results are shown after normalization to the values obtained for control HAECs (value = 1). Results are presented as means ± standard deviation (SD) from three independent experiments. Control (Ctr): untreated cells.

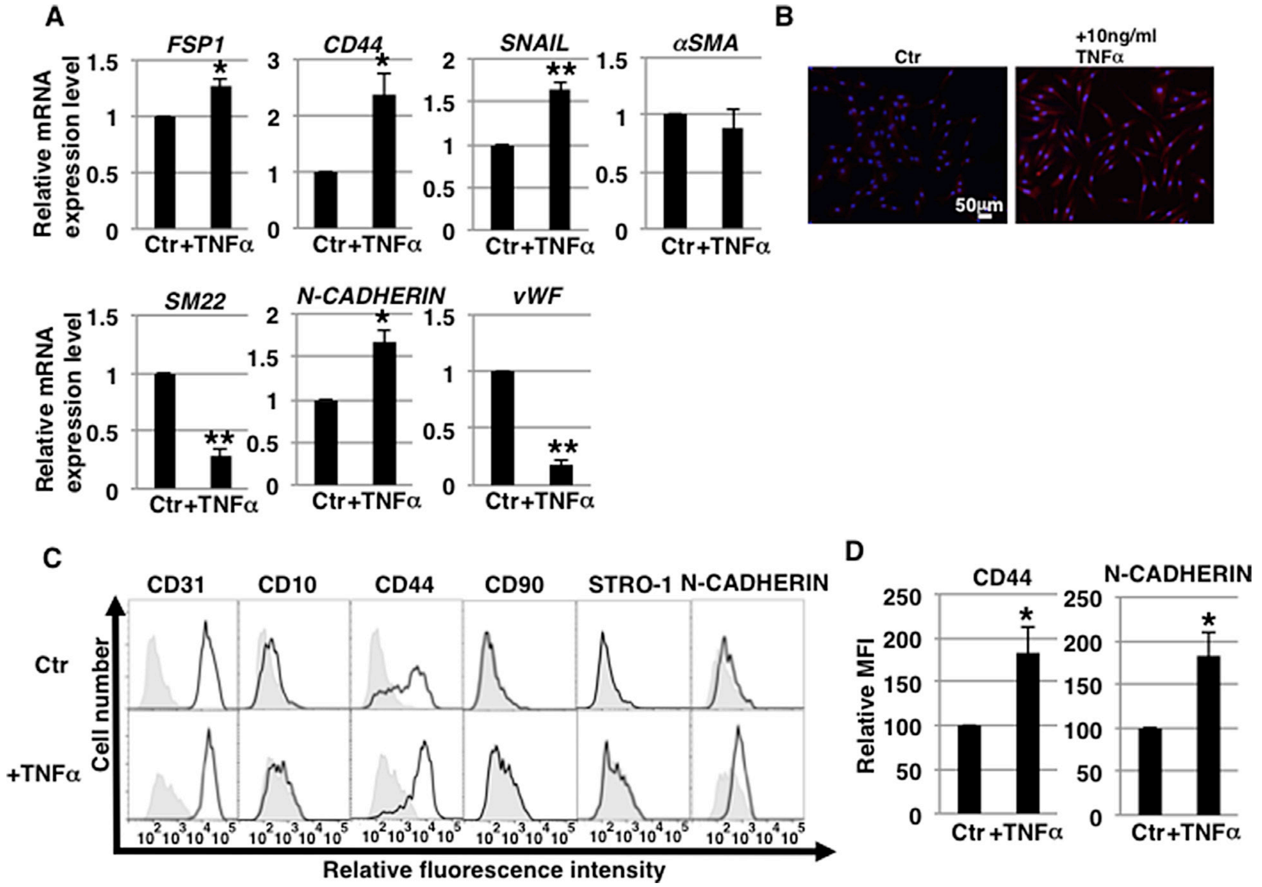

Supplementary Figure 4: EndMT-like differentiation is induced in 10 ng/ml TNFα-treated HAECs. (**A**) Real-time PCR analysis of EndMT markers (fibroblast marker, *FSP1*; mesenchymal markers, *CD44*, *SNAIL*, *N-CADHERIN* and a*SMA*; smooth muscle cell marker, *SM22*; endothelial marker, *vWF*) performed using cDNA derived from control and short-term (3 days) 10 ng/ml TNFα-treated HAECs. Results shown were normalized against values obtained for control HAECs (value = 1). Results are presented as means ± SD from three independent experiments. (**B**) Immunocytochemical staining of FSP1 performed in short-term (3 days) 10 ng/ml TNFα-treated HAECs. Representative images are shown (FSP1, *red*; DAPI, *blue*). (**C** and **D**) FACS analysis of cell surface markers (mesenchymal markers, CD44 and N-CADHERIN; mesenchymal stem cell markers, CD10, CD90 and STRO-1; endothelial marker, CD31) performed for short-term (3 days) 10 ng/ml TNFα-treated HAECs. Representative results are shown (C). Controls are depicted by *thin gray lines*. MFIs relative to control HAECs of three independent experiments are shown (D). $^{*}P < 0.05$; $^{**}P < 0.01$. Control (Ctr): untreated cells.

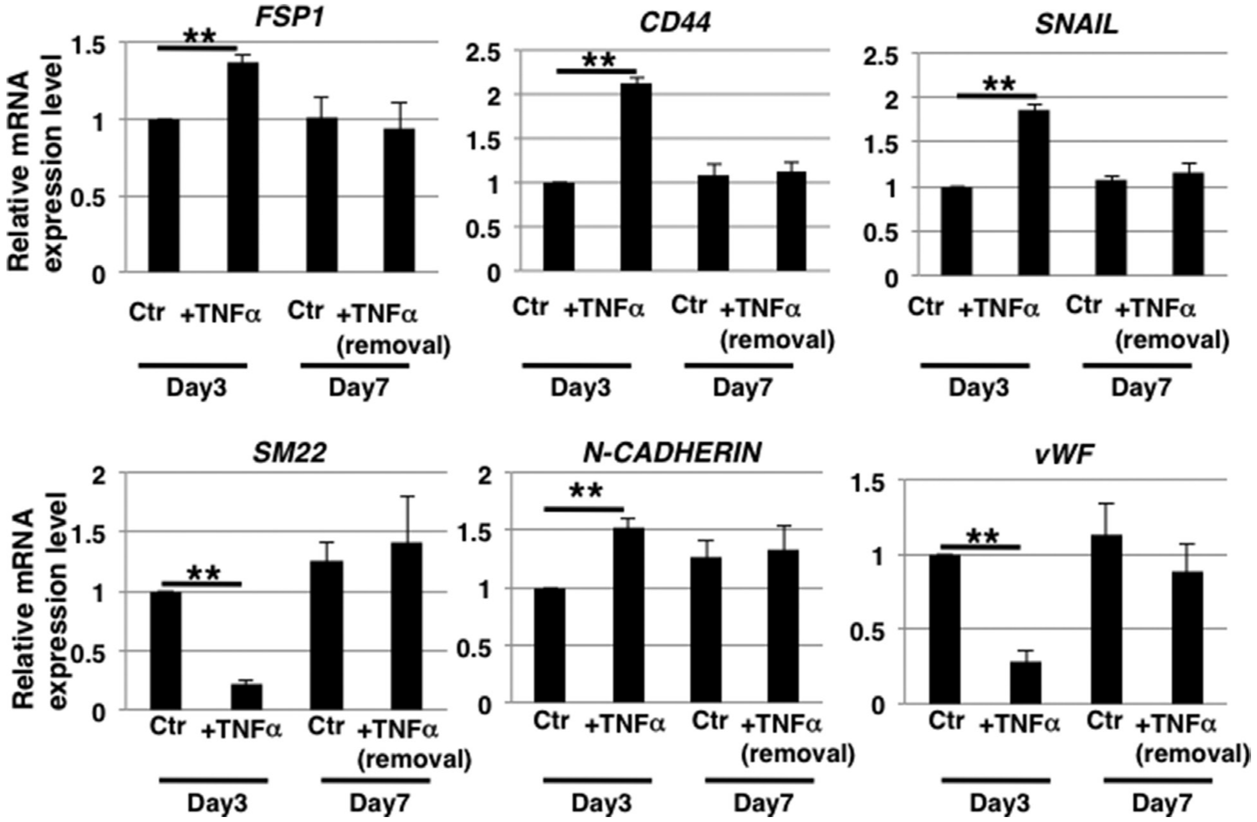

Supplementary Figure 5: EndMT-like differentiation in short-term (3 days) 10 ng/ml TNFα-treated HAECs is reversible. Real-time PCR analysis of markers for EndMT performed using cDNA derived from control, short-term (3 days) 10 ng/ml TNFα-treated HAECs and short-term (3 days) 10 ng/ml TNFα-treated HAECs 4 days after removal of TNFα. Results shown were normalized against values obtained for control HAECs (value = 1). Results are presented as means ± SD from three independent experiments. $^{**}P < 0.01$. Control (Ctr): untreated cells.

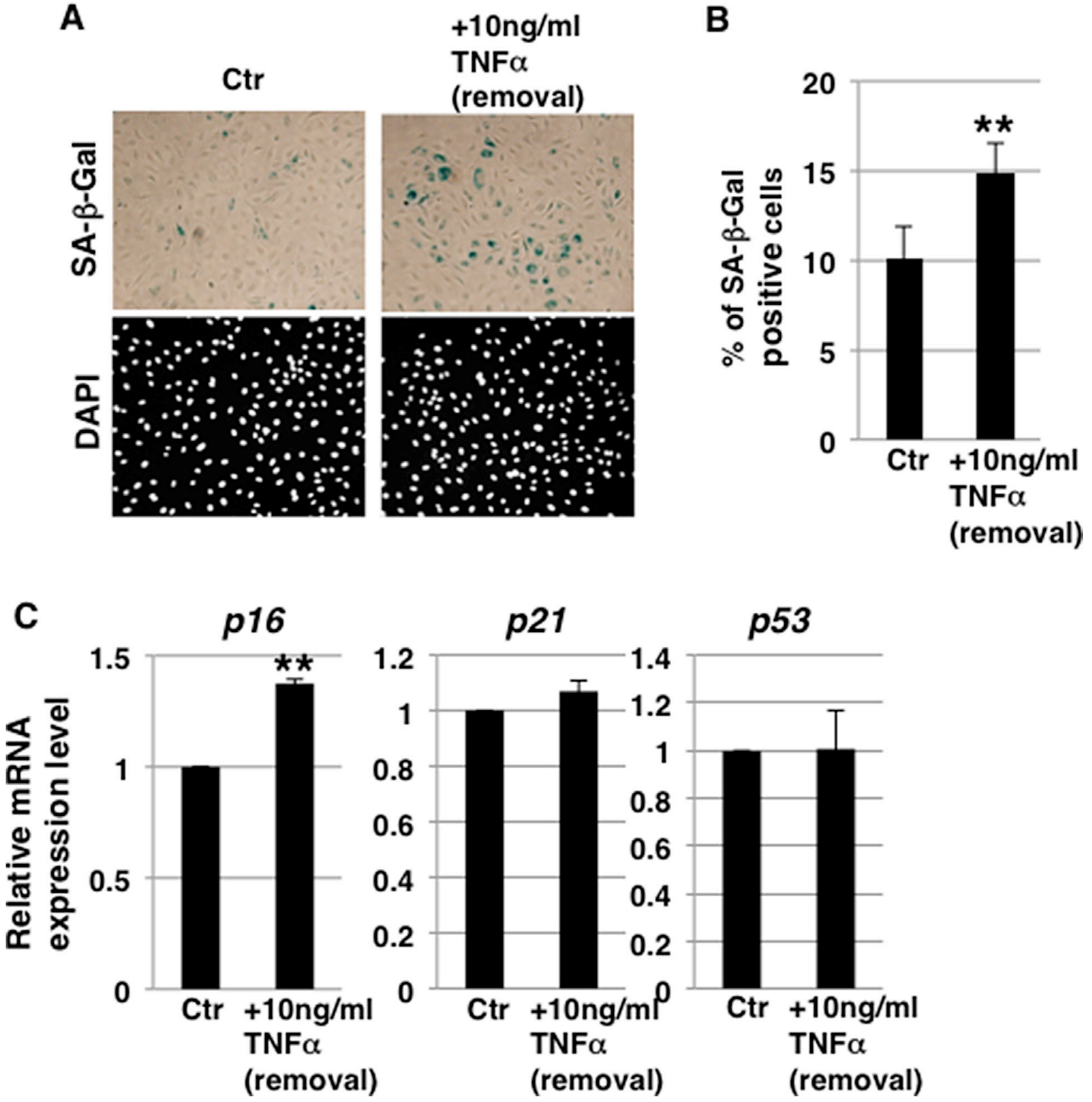

Supplementary Figure 6: HAECs reversed from EndMT-like differentiation show partial induction of premature senescence. (**A** and **B**) SA-β-Gal senescence assay performed in short-term (3 days) 10 ng/ml TNFα-treated HAECs 4 days after removal of TNFα (reversed HAECs). Cells were stained for SA-β-Gal activity and SA-β-Gal-positive cells were quantified as percentage of total cells. Representative images of SA-β-Gal and DAPI stainings are shown (A). Results are presented as means ± SD from four fields (B). (**C**) Real-time PCR analysis of senescence markers (*p16*, *p21* and *p53*) performed using cDNA derived from control and short-term (3 days) 10 ng/ml TNFα-treated HAECs 4 days after removal of TNFα. Results shown were normalized against values obtained for control HAECs (value = 1). $^{**}P < 0.01$. Control (Ctr): untreated cells.

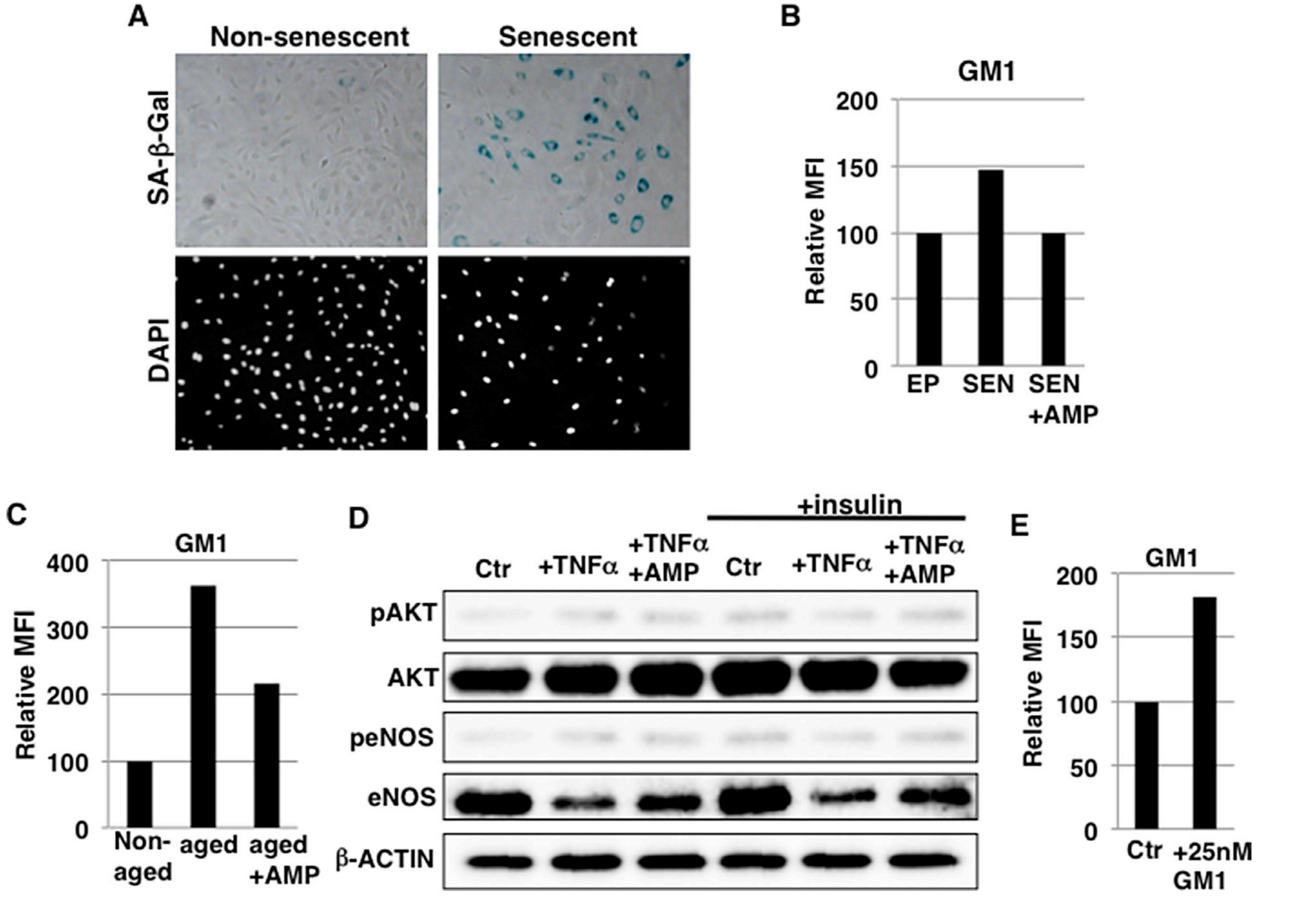

Supplementary Figure 7: GM1 increase in aged, senescent and exogenously GM1-overexpressing HAECs. (**A**) Non-senescent (EP) and senescent (SEN) HAECs stained for SA-β-Gal activity. Representative images are shown for SA-β-Gal and DAPI stainings. (**B**–**D**) Cell surface levels of GM1 in SEN-HAECs (B) and aged HAECs (C) with or without AMP-dNM treatment (3 days) and in non-aged HAECs. (D) Western blot analysis of insulin signaling performed in TNFα-treated aged HAECs with or without AMP-dNM treatment. (**E**) EP-HAECs cultured with or without 25 nM exogenous GM1 (24 h) analyzed by flow cytometry. MFIs relative to control HAECs are shown. Control (Ctr): untreated cells.
